# Automated diagnosis of anterior cruciate ligament via a weighted multi-view network

**DOI:** 10.3389/fbioe.2023.1268543

**Published:** 2023-10-10

**Authors:** Feng Li, Penghua Zhai, Chao Yang, Gong Feng, Ji Yang, Yi Yuan

**Affiliations:** ^1^ Orthopedic Department, Ningbo No. 2 Hospital, Ningbo, China; ^2^ Center for Pattern Recognition and Intelligent Medicine, Guoke Ningbo Life science and Health industry Research Institute, Ningbo, China

**Keywords:** multi-view learning, ACL diagnosis, computer-aided diagnosis, deep learning, magnetic resonance imaging

## Abstract

**Objective:** To build a three-dimensional (3D) deep learning-based computer-aided diagnosis (CAD) system and investigate its applicability for automatic detection of anterior cruciate ligament (ACL) of the knee joint in magnetic resonance imaging (MRI).

**Methods:** In this study, we develop a 3D weighted multi-view convolutional neural network by fusing different views of MRI to detect ACL. The network is evaluated on two MRI datasets, the in-house MRI-ACL dataset and the publicly available MRNet-v1.0 dataset. In the MRI-ACL dataset, the retrospective study collects 100 cases, and four views per patient are included. There are 50 ACL patients and 50 normal patients, respectively. The MRNet-v1.0 dataset contains 1,250 cases with three views, of which 208 are ACL patients, and the rest are normal or other abnormal patients.

**Results:** The area under the receiver operating characteristic curve (AUC) of the ACL diagnosis system is 97.00% and 92.86% at the optimal threshold for the MRI-ACL dataset and the MRNet-v1.0 dataset, respectively, indicating a high overall diagnostic accuracy. In comparison, the best AUC of the single-view diagnosis methods are 96.00% (MRI-ACL dataset) and 91.78% (MRNet-v1.0 dataset), and our method improves by about 1.00% and 1.08%. Furthermore, our method also improves by about 1.00% (MRI-ACL dataset) and 0.28% (MRNet-v1.0 dataset) compared with the multi-view network (i.e., MRNet).

**Conclusion:** The presented 3D weighted multi-view network achieves superior AUC in diagnosing ACL, not only in the in-house MRI-ACL dataset but also in the publicly available MRNet-v1.0 dataset, which demonstrates its clinical applicability for the automatic detection of ACL.

## 1 Introduction

The anterior cruciate ligament (ACL) is an autologous tissue structure with a large number of nerves and blood vessels that maintain the function of the knee (Gianotti et al., 2009; Bram et al., 2021). ACL tears are often caused by inappropriate exercise habits (Bourne et al., 2019; Montalvo et al., 2019; Grassi et al., 2020). ACL tears lead to decreased meniscus motility and degeneration and may increase the risk of an inflammatory response (Musahl and Karlsson, 2019; Fleming et al., 2022). Patients with severe ACL tears are even unable to walk and require replacement surgery to repair the knee joint (Ariel de Lima et al., 2021). Therefore, building an effective ACL computer-aided diagnosis (CAD) system and achieving timely and accurate clinical examination and diagnosis play an important role in the follow-up treatment and rehabilitation of patients.

Magnetic resonance imaging (MRI) has the advantages of high soft tissue resolution, multi-directional imaging, no radiation, and no pain (Zhao et al., 2020). MRI can clearly show the injury in various parts of the knee joint, thus helping clinicians to make diagnoses and treatments. Therefore, MRI is a widely accepted and used imaging technique for diagnosing ACL tears. However, radiologists need to spend a lot of time reading MRI scan slice by slice during the diagnostic process, which is prone to missed and false detections. Meanwhile, it may still be challenging for inexperienced radiologists to make an accurate diagnosis. Therefore, it is crucial to build a diagnostic system to assist physicians in achieving good pre-clinical prediction. The purpose of this study is to demonstrate the applicability of a fully automated diagnostic system to detect ACL tears.

In recent years, deep learning techniques have been well explored in medical image analysis, such as reading chest radiographs (Rajpurkar et al., 2018), chest CT (Xu et al., 2023) and brain MRI (Çinar and Yildirim, 2020). The advantage of deep learning lies in its ability to automatically learn sufficient semantic information from a large of samples to achieve classification, detection, and other tasks (Fourcade and Khonsari, 2019; Budd et al., 2021). Given the potential of deep learning, there is growing interest in applying it to the field of knee joint ACL diagnosis (Liu et al., 2019; Astuto et al., 2021; Jeon et al., 2021). However, there are some unique challenges with deep learning for MRI detection. First, it may be difficult to assess abnormalities on two-dimensional (2D) slices because the three-dimensional (3D) orientation of the ligament fibers is an important consideration when making a diagnosis. Second, single-view images may not fully reveal the overall morphology and features of the ACL. Finally, slight ACL tears may only occur in a small fraction of the entire 3D MRI volume. In response to these challenges, we build a 3D multi-view convolutional neural network-based diagnosis system and investigate its applicability for the automatic detection of ACL in the knee joint at MRI.

## 2 Materials and methods

### 2.1 Datasets

To demonstrate the applicability of our presented system for ACL diagnosis, we evaluate the system on two MRI datasets, one is the in-house MRI-ACL dataset and the other is the publicly available MRNet-v1.0 dataset^1^ (Bien et al., 2018). The type, the total number, the number of views and ACL patients, and the division of training and test sets for each dataset are summarized in Table 1. The details of the two datasets are as follows:

**TABLE 1:** Statistics of the in-house MRI-ACL dataset and the publicly available MRNet-v1.0 dataset.

Dataset	Type	Total number	View	ACL number	Number (train, test)
**MRI-ACL**	in-house	100	4	50	(80, 20)
**MRNet-v1.0**	public	1,250	3	208	(1,130, 120)

#### 2.1.1 MRI-ACL

Knee joint MRI cases are collected from a general hospital in Ningbo, China, from January 2021 to December 2021. The study is approved by the Ethics Committee of Ningbo No. 2 Hospital. All protected patient health information in the DICOM header is eliminated by data masking approaches, including patient name, institution ID, and referring physician name. We collect four MRI views for each case, namely, T1-sagittal, T2-sagittal, T2-coronal, and T2-transverse. The size of each slice is 512 × 512 and the number of slices ranges from 15 − 20. We divide all patients into ACL tears and normal according to the actual requirements of the hospital and MRNet-v1.0 (Bien et al., 2018). The distinguishing criterion is whether the patient has an ACL tear in the knee joint. To annotate the lesion as accurately as possible, a radiologist first annotates the MRI based on the annotation criterion and his experience. Then, the above annotations must be calibrated by a chief physician. We collect a total of 100 cases without patient overlap and four views per patient. We randomly select 70 cases for training, 10 cases for validation, and the remaining 20 cases as the test set.

#### 2.1.2 MRNet-v1.0

The dataset contains 1,370 knee MRI examinations that are released by Stanford University between January 2001 and December 2012, of which 120 samples are not available. The MRNet-v1.0 dataset is the largest publicly available annotated knee MRI dataset. All samples in the dataset contain three MRI views, sagittal plane T2-weighted series, coronal plane T1-weighted series, and axial plane PD-weighted series (Bien et al., 2018). The size of each image is 256 × 256 and the number of slices ranges between 17 − 61. Each case is labeled according to whether the patient has suffered an ACL tear, meniscal tear, or other knee joint abnormality. It should be noted that each examination may contain multiple labels, for example, if a case is labeled as positive for abnormality and ACL tear, it indicates that there are other forms of abnormality besides ACL tear. More details on the data can be found in the original paper. In our study, only ACL tears are considered as positive samples and other abnormal or normal patients are considered as negative samples. We train and test our presented model using the publicly available training set (1,130 scans) and test set (120 scans) with no patient overlaps, and the split refers to previous work (Unnikrishnan et al., 2021; Azcona et al., 2020). Meanwhile, of the 1,130 scans available for training, we randomly sample 20% as the validation set.

### 2.2 Method overview

**FIGURE 1:**
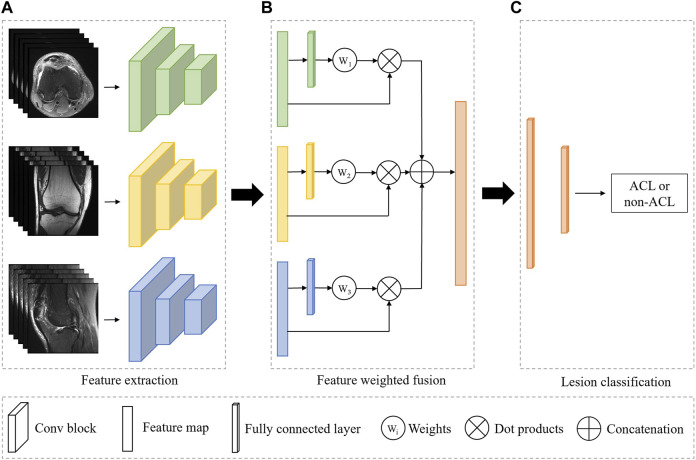
Overview of the fully automated deep learning-based ACL diagnosis system. The proposed system consists of three independent modules. **(A)** The feature extraction module is used to automatically extract features from the input image. **(B)** The feature weighted fusion module is used to combine the features of different views in a weighted manner. **(C)** The lesion classifier module is to identify the ACL from all patients. Green, yellow, and blue represent the different MRI views, and orange indicates the combined feature or the classifier.

**FIGURE 2:**
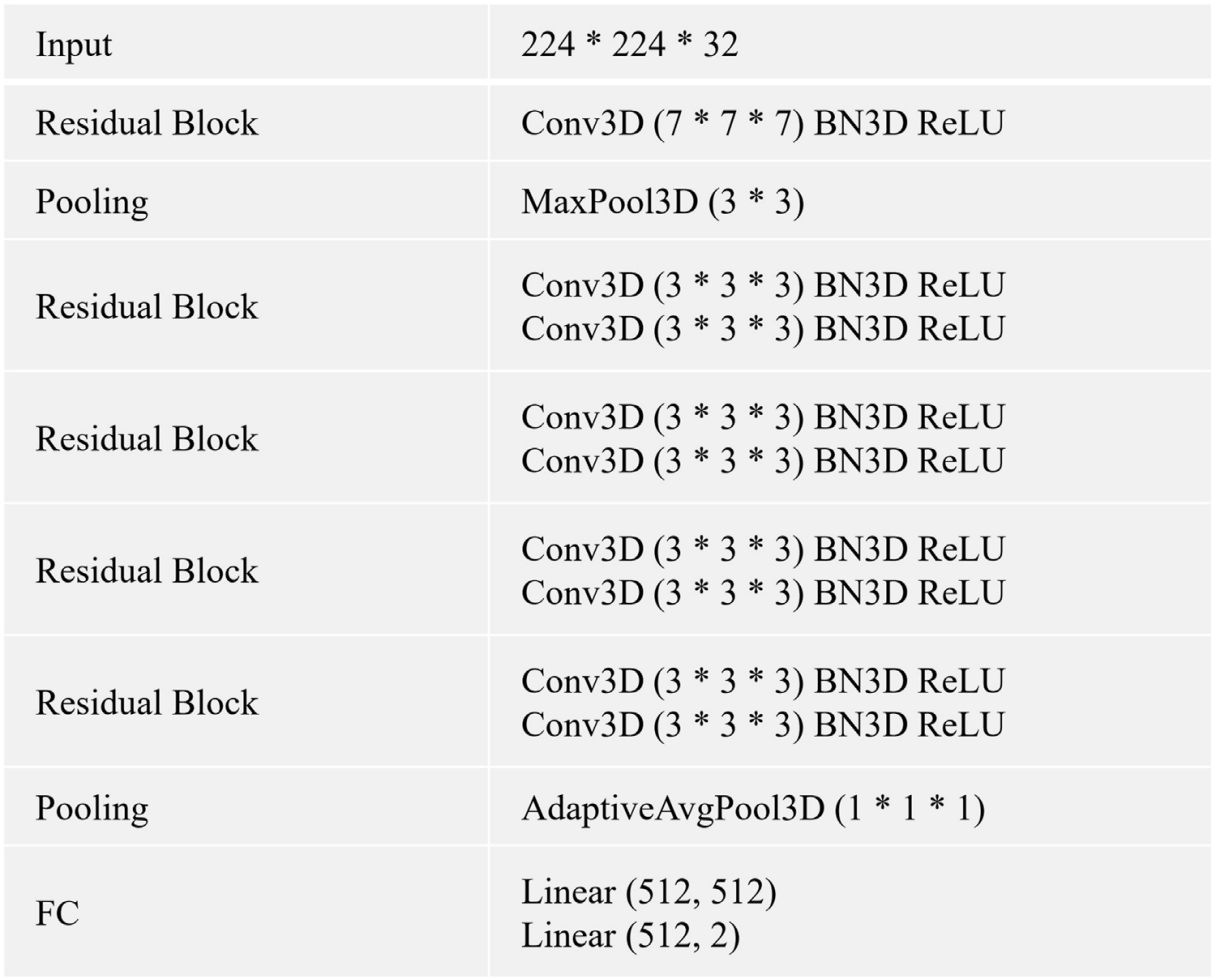
The CNN is built by using the listed layers from the top to the bottom. Specifically, BN3D, 3D batch normalization; Conv3D, 3D convolution; FC, fully connected layer; AdaptiveAvgPool3D, 3D adaptive average pooling; MaxPool3D, 3D maximum pooling; ReLU, rectified linear activation.

As shown in Figure 1, our system is based on a multi-view CNN to achieve ACL diagnosis by fusing features from different MRI views. The detailed architecture of the 3D CNN consists of residual-connected convolutional layers, pooling layers, activation function, and fully connected layer, as shown in Figure 2. Overall, our system consists of a feature extraction module, a feature weighted fusion module, and a lesion diagnosis module. The feature extraction module automatically maps the original knee joint MRI scans into features containing semantic information. The feature weighted fusion module weights and fuses all features according to the proportion of information provided by each view. The lesion diagnosis module maps the fused features to a final diagnostic score by a supervised classifier. We provide details of these modules in the following sections.

### 2.3 Feature extraction

The feature extraction module is a 3D CNN without fully connected layers for extracting MRI representations, as shown in Figure 1A. In our framework, all views share the weights of the model, and the advantages of shared weights are as follows: 1) Improving the training speed and efficiency, especially for 3D networks with more parameters and memory; 2) Improving the generalization ability of the model; 3) Improving interpretability, as it can learn complementary information from different views; 4) Reducing heterogeneity among different views.

Given a 3D MRI view $v\in R^{D\times H\times \times W}$ , where *D*, *H*, *W* represent the depth (number of slices), height, and width. We extract features by using residual blocks in the module, which is mathematically formulated as follows:

$$z=F\left(v,\left\{W_{i}\right\}\right)+v,$$ (1)

where *v* and *z* are the input and output vectors of a residual block. The function $F\left(x,\left\{W_{i}\right\}\right)$ represents the residual mapping. If the residual block has two layers, $F=W_{2}\sigma \left(W_{1}x\right)$ , where *σ* denotes ReLU activation function (Nair and Hinton, 2010). Finally, we can formulate the feature extraction process after all residual blocks as

$$h=f\left(x\right),$$ (2)

where *f* and *h* represent the feature extraction network (the stacked of all residual blocks) and learned view feature, respectively.

### 2.4 Feature weighted fusion

We simultaneously forward all MRI views of the same patient through a feature extraction encoder with shared weights to obtain their individual representations. To obtain the overall characteristic of the knee joint, we further employ a weighting mechanism to fuse these individual representations and map them into the same embedding space, as shown in Figure 1B. The purpose of this step is to embed the representations of all views into the same embedding space and reduce the gap between views. Suppose there are three views in an MRI sequence, namely, *v* = {*v*_1_, *v*_2_, *v*_3_}. Therefore, we can obtain the individual features *h* = {*h*_1_, *h*_2_, *h*_3_} after feature extraction module. To get the weight of each view, we first feed the features of all views separately into a multi-layer perceptron (MLP), thus, the MLP will output three weight values. Next, we apply a softmax function to normalize these weights. Finally, the representations of each view are weighted and concatenated to form a unified visual feature.

### 2.5 ACL diagnosis

We can regard the final lesion diagnosis task as a binary classification task, i.e., separating ACL patients from all patients. Therefore, we use a binary classifier to map the fused representations to the final diagnostic score, as shown in Figure 1C. The classifier is based on a two-layer MLP. Assuming that there are *n* views and the feature dimension of each view obtained by the feature extraction module is 512, and the dimension of the concatenated feature is *n* × 512. The first layer of the MLP maps the feature to the dimension of 512, and the second layer will output the binary classification result.

During the training process, we use the cross-entropy loss to measure the training effect of the model. The cross-entropy loss formula is as in Eq. 3.

$$L_{y,y^{ˆ}}=-\left[ylogyˆ+\left(1-y\right)log\left(1-yˆ\right)\right],$$ (3)

where *y* is the ground truth, *y* = 1 if a patient suffers from an ACL tear, otherwise *y* = 0. The *yˆ* represents the prediction of the model.

### 2.6 Implementation details

In this study, we use 3D CNN (He et al., 2016) as the baselines for all experiments. We describe the architecture and details of 3D CNN in Figure 2. The method is trained using an Adam optimizer. Meanwhile, we train the model for 100 epochs with an initial learning rate of 0.0001 and a batch size of 32. The momentum and weight decay coefficient are set to 0.9 and 0.0001, respectively. Throughout the training phase, the model that achieves the best performance on the validation set is used for the evaluation. We implement our method with PyTorch using NVIDIA Tesla A100 40 GB GPUs. We use the area under the receiver operating characteristic curve (AUC) to evaluate all methods.

## 3 Results and experiments

### 3.1 Comparison with existing methods

In this section, we compare the diagnosis performance of our presented system with several existing methods on the in-house MRI-ACL dataset and the publicly available MRNet-v1.0 dataset, as shown in Table 2. We divide the existing methods into two categories, one is single-view methods and the other is multi-view methods. The former uses only one view to train and evaluate the model, represented by the method of training 3D VGG16 (Simonyan and Zisserman, 2014) and 3D ResNet-10 (He et al., 2016) from scratch, respectively. The latter achieves ACL diagnosis by fusing different views, including MVCNN (Su et al., 2015) and MRNet (Bien et al., 2018). To perform a fair comparison, we use the same initial learning rate and image size as our model for all the above models. The AUC is used to evaluate the performance of ACL diagnosis comprehensively. As can be seen from Table 2, our system outperforms 3D VGG16, 3D ResNet-10, MVCNN, and MRNet on the MRI-ACL dataset by 3.00%, 1.00%, 2.00%, and 1.00%, respectively. Our system also achieves the best AUC of 92.86% on the MRNet-v1.0 dataset, which exceeds 3D VGG16, ResNet-10, MVCNN, and MRNet by 2.61%, 1.08%, 0.92%, and 0.28%, respectively. The results show that our method outperforms existing single-view and multi-view classification models and can solve the ACL diagnosis task well.

**TABLE 2:** Comparison with existing methods on MRI-ACL and MRNet-v1.0 datasets.

Methods	MRI-ACL	MRNet-v1.0
3D VGG16 Simonyan and Zisserman, (2014)	94.00	90.25
3D ResNet-10 He et al, (2016)	96.00	91.78
MVCNN Su et al, (2015)	95.00	91.94
MRNet Bien et al. (2018)	96.00	92.58
Ours	97.00	92.86

### 3.2 Ablation for view number

**FIGURE 3:**
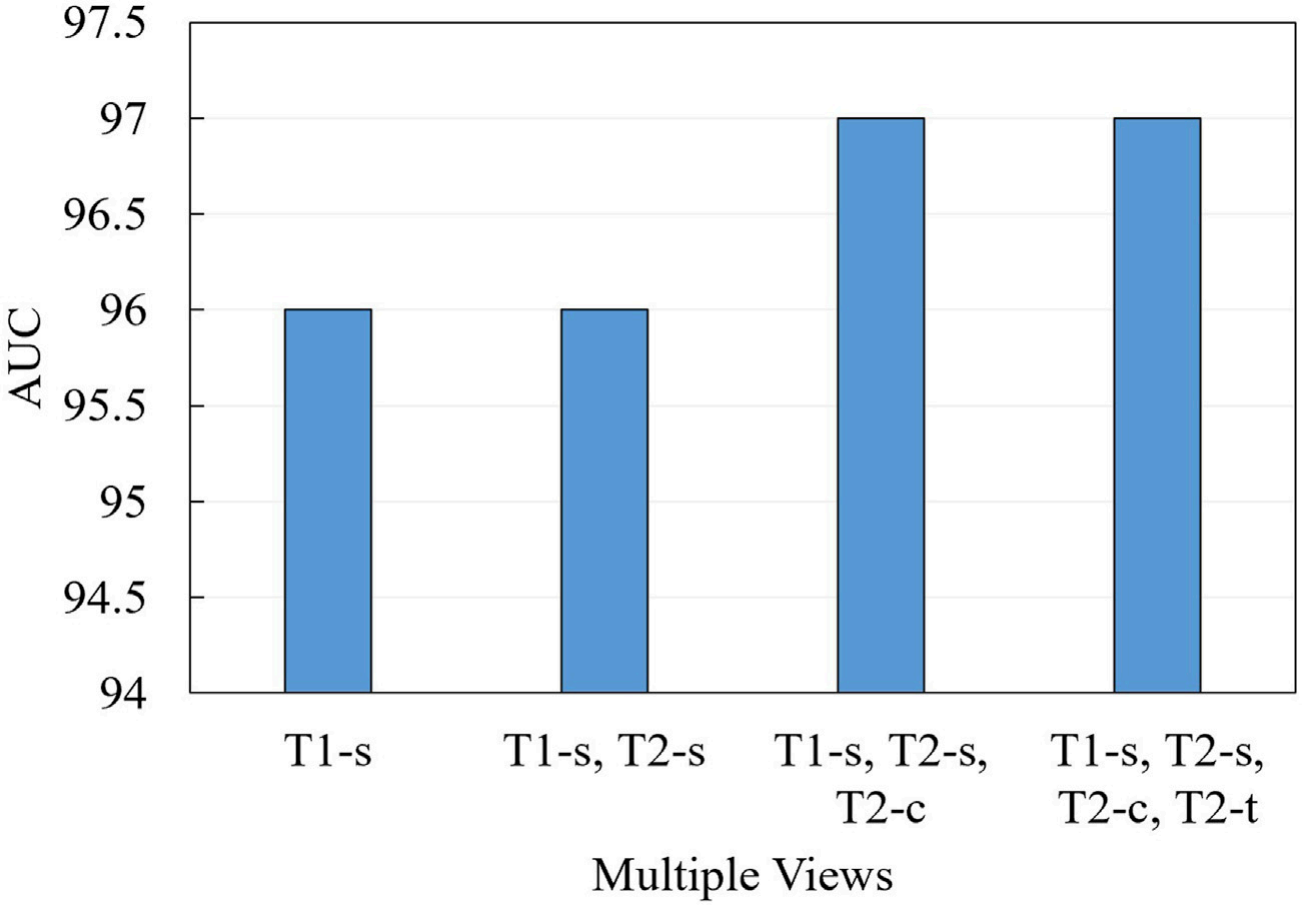
Ablation for the number of views on MRI-ACL dataset.

**FIGURE 4:**
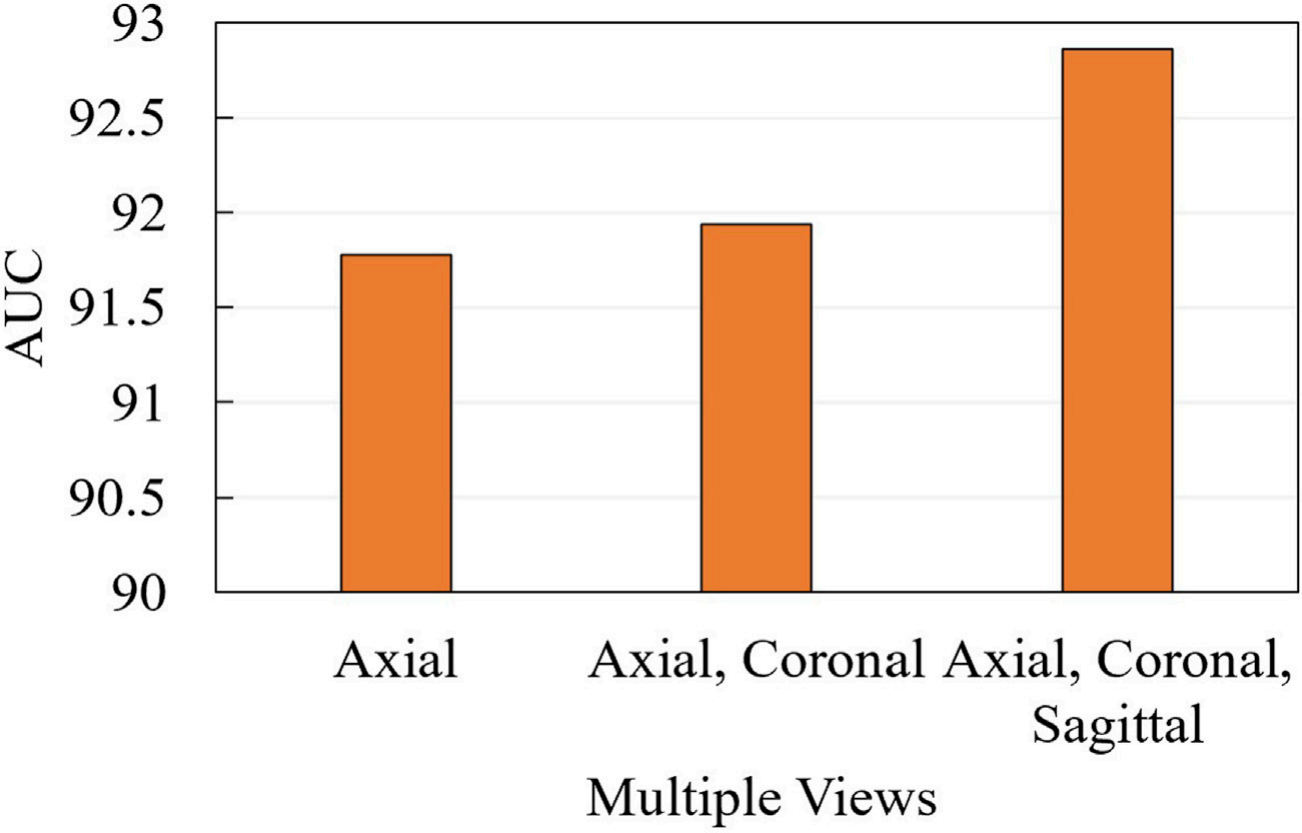
Ablation for the number of views on MRNet-v1.0 dataset.

We further conduct ablation experiments on the MRI-ACL dataset and MRNet-v1.0 dataset to evaluate the effectiveness of the presented system with different numbers of views, as shown in Figure 3 and Figure 4, respectively. If we only use one view, the best AUC is 96.00%, which is 1.00% lower than the system based on four views on the MRI-ACL dataset. Meanwhile, the system achieves the best AUC of 91.78% using a single view, which is 1.08% lower than the system using three views on the MRNet-v1.0 dataset. From Figures 3, 4, It can be seen that the AUC improves as the number of views increases. Therefore, we can observe that multiple views contribute positively to ACL diagnosis.

### 3.3 Ablation for fusion

**FIGURE 5:**
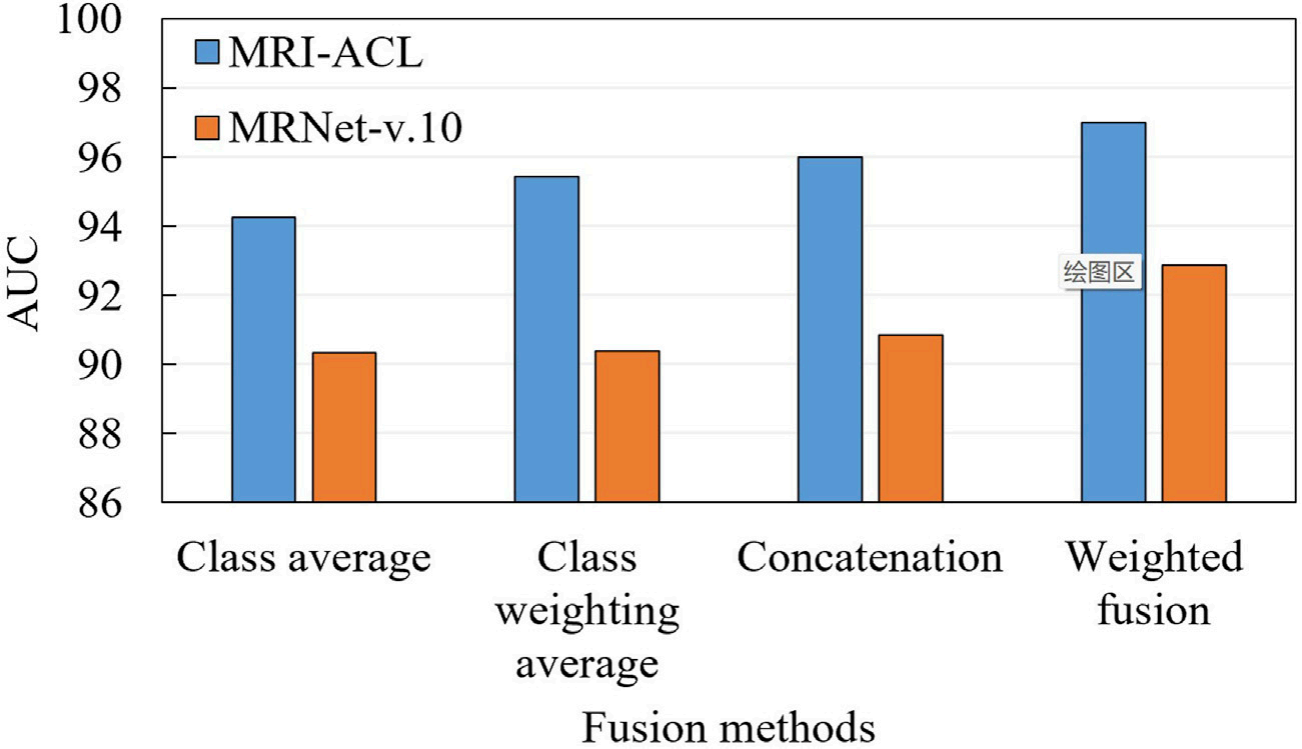
Ablation for fusion on MRI-ACL and MRNet-v1.0 datasets.

To demonstrate the effectiveness of the fusion approach, we compare two typical fusion approaches, including label fusion (contains a class average method and a class probability weighting method) and feature fusion (i.e., feature concatenation and our presented method), as shown in Figure 5. The label fusion occurs on the prediction results, which are calculated by averaging or weighted average over all the results. Feature fusion happens at the representation generation stage. First, each view generates its own representation through the backbone. Later, these representations are concatenated or weighted concatenated into the embedding space. The concatenated representation is fed into a classifier to obtain the final ACL diagnosis scores. From Figure 5, We can observe three aspects: 1) Compared with the two fusion methods, the result of the feature fusion is better than label fusion; 2) The weighted fusion is better than the direct fusion method; 3) Our method achieves the best results with an AUC of 97.00% (MRI-ACL dataset) and 92.86% (MRNet-v1.0 dataset), respectively.

## 4 Discussion

In this study, we build a knee joint ACL diagnosis system based on a 3D multi-view convolutional neural network, which provides a fully automated model for completing knee joint assessment based on multiple views of MRI. We also demonstrate the applicability of ACL automatic detection in the knee joint. The experimental results show that the high AUC of the presented ACL diagnosis system can assist radiologists in reading images to improve diagnostic accuracy and reliability. ACL tears are prevalent worldwide, especially with increased incidence due to sports injuries or aging. Our system is able to provide patients with a timely and accurate diagnosis of ACL. Accurate diagnosis of ACL is the key to treatment and rehabilitation. Therefore, this study has important clinical significance.

There are several systems based on deep learning to help radiologists detect and analyze specific tissue lesions with good performance and applications, such as chest radiograph detection (Rajpurkar et al., 2018; Zhou et al., 2022) and organ segmentation (Gibson et al., 2018; Wang et al., 2019). In addition, researchers have also made some progress in ACL diagnosis. However, existing automatic assessment systems for knee joint lesions are mostly based on 2D neural networks or single-view images. To investigate the feasibility of deep learning for ACL tear detection on MRI, Liu et al. (Liu et al., 2019) first localizes the ACL by using two deep CNNs, and then detect tears within the ligament based on a CNN. The AUC of the method for detecting ACL tears is 98%. Their method outperforms most existing methods and our presented method in AUC. However, their method requires a knee joint localization stage. Therefore, there are three weaknesses: 1) radiologists need a lot of time and effort to annotate the knee joint location, 2) ACL diagnosis results depend on localization results, and 3) training time and model parameters are increased. We perform ACL diagnosis based on the 3D anatomical structure of raw MRI with an AUC of 97.00% and do not require joint location annotations. Bien et al. (Bien et al., 2018) detect ACL tears based on MRNet in the MRNet-v1.0 dataset with an AUC of 92.58%, which is a binary classifier based on a 2D multi-view convolutional neural network. Although MRNet also uses multiple views, it only implements a simple concatenation of features. Compared with MRNet, our method is based on a 3D network and feature weighted fusion, achieving a better AUC of 92.86%. Our approach is more in line with the reading process of radiologists. Astuto et al. (Astuto et al., 2021) develop a 3D CNN to detect lesions in MRI, as well as grade abnormalities in cartilage, bone marrow, menisci, and ACL. They achieve an AUC of 90.00% in identifying ACL tears. Although they learn the 3D characteristics of MRI, they only build a single-view model and could not fully explore the complementary information between the different views. Overall, our presented system is not only able to fuse information from different views but also fully learn the 3D properties of lesions.

Although our presented system achieves excellent results, there are some limitations. First, although we have validated our method on the largest available ACL dataset, the dataset we collected and labeled is relatively small. Small datasets may affect the generalization performance of our model. Therefore, we need to further improve the robustness of the diagnostic system with larger training datasets, data augmentation approaches, and transfer learning. Second, we only label knee joint ACL tears, lacking annotations for other lesions. Although our system has excellent performance, multi-task learning may allow the system to learn more detailed information about the image, which helps to improve the diagnostic accuracy of different lesions. Finally, there is one limitation of CNN is that it is still a black-box approach, which makes it difficult to interpret which features are processed by the network. If we want to provide better assistance to physicians and patients, we need to further study and address the problem of interpretability.

In summary, our study demonstrates the applicability of a 3D deep learning-based approach for the automatic detection of knee joint ACL tears on MRI. Meanwhile, experimental results on different datasets demonstrate that fusing multiple views of MRI can greatly improve diagnostic accuracy. This study is an important exploration of advancing artificial intelligence methods to assist radiologists in medical image analysis. It is beneficial to the development and application of computer-aided diagnosis systems in clinics in the future.

## 5 Conclusion

In this study, we present a 3D deep learning-based diagnosis system to automatically detect ACL tears by fusing various MRI views of the same patient. We evaluate the system on two ACL datasets, namely, the in-house MRI-ACL dataset and the publicly available MRNet-v1.0 dataset. Experimental results show that the method is superior or comparable to existing single-view models and multi-view models, demonstrating the clinical effectiveness and applicability of the presented method.

## Data Availability

The datasets presented in this article are not readily available because the data that support the findings of this study are available on reasonable request from the authors. The imaging data were not publicly available because of restrictions (containing information that could compromise the privacy of research participants). Requests to access the datasets should be directed to nbjoint@163.com.

## Footnotes

1
https :// stanfordmlgroup.github.io /competitions/ mrnet /

## Abbreviations

ACL, anterior cruciate ligament; MRI, magnetic resonance imaging; 3D, three-dimensional; 2D, two-dimensional; CNN, convolutional neural network; AUC, area under the receiver operating characteristic curve.
